# Kitchen Medicine

**Published:** 1874-04

**Authors:** J. Solis Cohen


KITCHEN MEDICINE.—*By J. Solis Cohen, M.D.*—The potent remedial virtues of drugs are by no means to be ignored. Instances are frequent enough in which it is impossible to treat disease without their aid. Still, it is well now and then to have our attention directed to agents which are apt to be overlooked because they are so familiar, and undervalued because they are easy of access.

Diluents are often called for in the practice of medicine, and many of the articles used for this purpose owe most of their benefit to the water in which they are administered; and water alone, impregnated, it may be, with some kitchen remedy—as currant-jelly, or even barley, for example, in simple erythematous sore throat—will be just as valuable in many instances as a pharmaceutic extract.

Emetics are often called for, and in many cases mustard-and-water is just as valuable as sulphate of zinc or copper, or tartar-emetic, and so on, and sometimes more so.

In cases of severe cramp in the intestines, dry heat, applied from the door of a range or furnace, taken off and wrapped in folds of flannel to protect the skin, will often give prompter relief than a dose of opium. The mustard-plaster, so much in vogue and so very efficient as a topical remedy in many affections, is a familiar example of the value of kitchen medicine.

The use of table-salt in restraining hæmoptysis, and even the use of oil of turpentine, may be instanced as further examples. Cayenne pepper will do as good service in certain affections, as in bowel-complaints, or used as a gargle in sore-throat, or taken in spirits of some kind as a preventive of the deleterious effects of exposure to malaria, as many other remedies more frequently prescribed. Vinegar comes in use as a local application very often; it is good to impregnate a sponge both to allay excessive action in the skin in fevers and in the sweats of hectic. It is often as good internally, in some form or other, as are other acids, mineral as well as vegetable, to assist digestion in certain varieties of dyspepsia. It is often as good an ingredient in a gargle as any other acid, and very often the best inhalation that can be given in certain inflammatory conditions of the respiratory passages. Salt-and-water is an excellent tonic to the skin; an efficient laxative in moderate costiveness, judiciously managed, better often than any saline purge; the very best and least injurious substance to use in the nasal douche to detach the accumulated secretions of certain forms of catarrhal inflammations of the nasal passages. Spices of various kinds are splendid carminatives internally administered, and, quilted between folds of flannel, they form an excellent bandage to be worn over the abdomen of patients subject to bowel-complaints. Dry salt similarly quilted between folds of flannel, and heated before a fire, retains heat a long time, and is an excellent application in painful conditions of the bowel.

Cucumbers, cabbage, tomatoes, fruits of various kinds properly selected, unbolted flour, milk, calves' brains, sweet-breads, oysters, terrapins, certain kinds of fish, and other articles of ordinary or occasional diet, contain certain ingredients, in far better elaboration than the skill of the pharmacist can prepare, which can often be utilized for supplying deficiencies in the economy of the treatment of chronic disease.

Milk, lard and sweet-oil, applied to bruises, to harsh skins, or used for anointing the body in scarlet fever and the like, are often of the greatest value. In chronic ulcerative laryngitis entailing dysphagia, the preliminary deglutition of a little sweet-oil will often protect the parts from the mechanical irritation of food, and render swallowing more efficient and less painful.

